# Bodybuilding championships and myotonia congenita

**Published:** 2016-07-06

**Authors:** Mohammad Rohani, Shahnaz Miri, Alireza Rezai-Ashtiani

**Affiliations:** 1 Department of Neurology, School of Medicine, Iran University of Medical Sciences, Tehran, Iran; 2 Department of Medicine, Medstar Union Memorial Hospital, Baltimore, Maryland, United States; 3 Department of Neurology, School of Medicine, Arak University of Medical Sciences, Arak, Iran

**Keywords:** Myotonia Congenita, Bodybuilding, Muscle Hypertrophy

A 25-year-old man presented with a 10-year history of difficulty in relaxing his muscles. He was bodybuilding champion in his city without doing any exercise. 

Neurologic examination revealed well-formed skeletal muscles (first part of the video: http://ijnl.tums.ac.ir/public/891-725-1-Part1.mov) and myotonia most prominent in the eyes (a lag in opening the eyes after forceful closure) and hands (delayed hand opening after gripping) (second part of the video: http://ijnl.tums.ac.ir/public/891-726-1-Part2.mov). 

There was percussion myotonia in thenar muscles without prominent muscle weakness. Electromyogram showed myotonic discharges.

Myotonia congenita is a rare hereditary neuromuscular channelopathy characterized by delayed relaxation of skeletal muscles following voluntary contraction, beginning in the first or second decade of the life. It can be associated with muscle hypertrophy, stiffness, transient weakness, or cramping.^1^^,^^2^ Only patients with symptomatic myotonia require treatment with medications such as phenytoin, carbamazepine, or procainamide to reduce the excitability of the muscle membrane.

**Legends to the video**

First part of the video shows hypertrophic muscles of the arms and shoulder girdles; the second part shows delayed opening and relaxing the hands after gripping; and the third part shows myotonic discharges on electromyography.

## Notes:

**How to cite this article:** Rohani M, Miri S, Rezai-Ashtiani A. Bodybuilding championships and myotonia congenita. Iran J Neurol 2016; 15(3): 182.
